# Genes encoding equine β-lactoglobulin (*LGB1* and *LGB2*): Polymorphism, expression, and impact on milk composition

**DOI:** 10.1371/journal.pone.0232066

**Published:** 2020-04-22

**Authors:** Lukasz Wodas, Mariusz Mackowski, Alicja Borowska, Kamila Puppel, Beata Kuczynska, Jakub Cieslak

**Affiliations:** 1 Department of Horse Breeding, Poznan University of Life Sciences, Poznan, Poland; 2 Horse Genetic Markers Laboratory, Poznan University of Life Sciences, Poznan, Poland; 3 Cattle Breeding Division, Department of Animal Science, Warsaw University of Life Sciences, Warsaw, Poland; INIA, SPAIN

## Abstract

β-lactoglobulin is one of the most abundant milk whey proteins in many mammal species, including the domestic horse. The aim of this study was to screen for polymorphism in the equine *LGB1* and *LGB2* gene sequences (all exons, introns, and 5’-flanking region) and to assess potential relationship of particular genotypes with gene expression levels (measured in milk somatic cells) and milk composition traits (protein, fat, lactose, and total β-lactoglobulin content). Direct DNA sequencing analysis was performed for twelve horse breeds: Polish Primitive Horse (PPH), Polish Coldblood Horse (PCH), Polish Warmblood Horse (PWH), Silesian, Hucul, Fjording, Haflinger, Shetland Pony, Welsh Pony, Arabian, Thoroughbred, and Percheron—and revealed the presence of 83 polymorphic sites (47 and 36 for *LGB1* and *LGB2* genes, respectively), including eight that were previously unknown. Association analysis of the selected polymorphisms, gene expression, and milk composition traits (conducted for the PPH, PCH, and PWH breeds) showed several statistically significant relationships; for example, the two linked *LGB1* SNPs (rs1143515669 and rs1144647991) were associated with total milk protein content (p < 0.01). Our study also confirmed that horse breed had significant impact on both gene transcript levels (p < 0.01) and on milk LGB content (p < 0.05), whereas an influence of lactation period was seen only for gene relative mRNA abundances (p < 0.01).

## Introduction

Ruminants, including cattle, sheep, and goats, are the major source of the milk produced and consumed all over the world. However at present, with the promotion of functional foods, there has been increased interest in milk from so-called minor dairy species. Special attention has been paid to mare’s milk, mainly on account of the similarity of its composition to human breast milk, its numerous health-promoting properties, and its low allergenicity. It is estimated that around 30 million people consume mare milk regularly, predominantly in Asia, where the tradition of kumis production continues to this day (such as Mongolia and Kazakhstan), but also in some European countries (such as Germany and France) [1]. Equine milk has a higher degree of compositional similarity to human breast milk than the milk of ruminants, and is thus considered a potential nutritional substitute for human infants [2]. Mare’s milk has less fat and protein, but higher lactose concentration, than cow’s milk [3,4]. The protein fraction of equine milk contains low levels of caseins, a class of proteins that predominates in the milk of ruminants [4,5]. Due to equine milk’s relatively low allergenic potential compared to cow, sheep, and goat milk, it would seem to be a valuable alternative for people suffering from hypersensitivity to the proteins found in ruminants’ milk, which is one of the most common food allergies globally [6]. Furthermore, because mare’s milk contains high levels of bioactive substances, such as lysozyme and lactoferrin, it can be used as a supportive treatment for some human diseases, like gastric ulcers and atopic dermatitis [7,8].

The most abundant components of the predominant whey protein fraction of mare’s milk are β-lactoglobulin (LGB) and α-lactalbumin (LALBA). Together, these proteins comprise about 60% of total equine milk whey protein [3]. β-lactoglobulin, the main subject of this study, is a globular protein found in the milk of various mammals, though not in that of humans, camels, lagomorphs, or rodents [5]. Although LGB was discovered over 80 years ago, its function remains unclear [9]. It is assumed that β-lactoglobulin plays a role in the transport of various small molecules; it may facilitate the uptake of lipophilic vitamins and modify enzyme activity. Similarly to the bovine protein, there are two isoforms of equine LGB, named LGB1 and LGB2. However, whereas the bovine β-lactoglobulin forms are the result of missense polymorphisms occurring within the same gene [10], domestic horses—like donkeys and dogs—have two separate paralogous genes encoding LGB1 and LGB2 [11]. Both genes are located on chromosome 25 (ECA25) and consist of seven exons. The recently updated horse genome assembly (EquCab 3.0) indicates that the total physical lengths of *LGB1* and *LGB2* are 4739 bp and 4816 bp, respectively (GenBank NC_009168.3). The equine LGB1 protein is made up of 162 amino acids, just like bovine LGB, while LGB2 has 163 aa residues. The predicted molecular mass of both forms is 18.5 kDa (LGB1) and 18.3 kDa (LGB2). The mean level of β-lactoglobulin in mare’s milk is 2.55 g kg^-1^, which is generally lower than in cow’s milk (~3.2 g kg^-1^) [4]. However, some different values have been found by different investigations. For example, Summer et al. (2005) [12] found the mean concentration of LGB in equine milk to be 2.8 g L^-1^, while Markiewicz-Kęszycka et al. (2013) [13] recorded a slightly lower value (2.6 g L^-1^).

The vast majority of studies to date on the effect of genetic variants on milk yield and composition have been conducted for cattle [14–17] and small ruminants [18,19]. Knowledge of the impact of DNA polymorphisms on domestic horse milk composition is very limited. Of the genes encoding equine whey proteins, only LYZ (lysozyme), LTF (lactoferrin), and LALBA (α-lactalbumin) have been “functionally” studied in the context of the influence of particular genetic variants on gene expression or milk composition [20,21]. Such studies are important mainly in the search for genetic markers associated with milk yield and composition, and to better understand the molecular mechanisms related to the regulation of milk protein gene expression. As β-lactoglobulin is one of the most abundant milk proteins, the encoding genes are interesting candidates for mammalian milk composition and production traits. Previous studies of Romanian Simmental cattle have suggested an association between *LGB* genotypes and milk yield [17]. Molee et al. (2015) [22] have shown that the specific combination of genotypes at the *GH* (growth hormone) and *LGB loci* can affect milk production and protein content and can therefore serve as a useful genetic marker in selection programs for crossbred Holstein cattle. Finally, it was suggested that the *LGB* gene may also be important in the context of milk processing technology, as particular genotypes are significantly associated with rennet coagulation time in Brown Swiss cows [23].

Current knowledge of equine *LGB1* and *LGB2* genes is limited. A recent study by Brinkmann et al. (2016) [11] of both gene coding sequences found only one polymorphic site in *LGB1* (a fragment encoding a signal peptide) and eight nonsynonymous variants in the *LGB2* gene (ten different protein variants were predicted). However to our knowledge, there has been no study focusing on the full nucleotide sequences of both genes (including introns and regulatory regions) aimed at finding the potential association between the detected genetic variants, gene expression, and the composition of mare’s milk. The main aim of this study was thus to screen for polymorphisms in the structural parts (exons and introns) and the 5’-flanking regions of the equine *LGB1* and *LGB2* genes, and to analyze their expression in different horse breeds at three lactation stages (the fifth, tenth, and fifteenth weeks after foaling). The results were compared to determine the potential relationships between the detected genetic variants, gene expression, and selected milk composition traits.

## Material and methods

### Identification and genotyping of genetic variants

Initial detection of the variants involved 96 genomic DNA samples representing animals (both genders) belonging to twelve different horse breeds: Polish Primitive Horse (n = 8), Polish Coldblood Horse (n = 8), Polish Warmblood Horse (n = 8), Silesian (n = 8), Hucul (n = 8), Fjording (n = 8), Haflinger (n = 8), Shetland Pony (n = 8), Welsh Pony (n = 8), Arabian (n = 8), Thoroughbred (n = 8), and Percheron (n = 8). All DNA samples used in this study derived from the Horse Genetic Markers Laboratory collection (Poznan University of Life Sciences, Poland), where they had previously been used for routine parentage control analyses.

Screening for polymorphisms included all exons, introns, and about 1000 bp of the equine *LGB1* and *LGB2* genes 5’-flanking regions. PCR primer sequence pairs were designed using the *Primer3* tool [24] based on the equine *LGB1* and *LGB2* genes nucleotide sequence (GenBank NC_009168.2). Altogether, eleven and twelve overlapping PCR primers pairs, for the *LGB1* and *LGB2* genes respectively, were synthesized by Sigma-Aldrich (Germany). In total, the analysis spanned 6915 bp and 6845 bp of the *LGB1* and *LGB2* genes respectively.

PCR amplification was carried out in a T-100 thermocycler (Bio-Rad, USA) under the following conditions: initial denaturation (95°C, 5 min); 35 cycles of denaturation (95°C, 1 min), primer annealing (temperature established experimentally, 1 min), elongation (72°C, 1 min); and final synthesis (72°C, 10 min). The amplified DNA samples were stored at 4°C until further analysis. The PCR products specificity was tested by electrophoresis (120 V, 30 min) in 1.5% agarose gel stained with ethidium bromide. Primer sequences and other amplification details for all investigated fragments are shown in S1 Table.

Prior to the DNA sequencing, PCR products were cleaned of unused primers and nucleotides with Exonuclease I and Thermosensitive Alkaline Phosphatase digestion (Thermo Fisher Scientific, USA). The following incubation conditions were used: 37°C, 30 min; 80°C, 15 min. The sequencing reaction, based on the BigDye Terminator v3.1 Cycle Sequencing Kit (Thermo Fisher Scientific, USA), was carried out in the T-100 thermocycler (Bio-Rad, USA) using the following protocol: initial denaturation (95°C, 5 min); 25 cycles of denaturation (95°C, 30 s), primer annealing (50°C, 10 s), and DNA synthesis (60°C, 4 min). Samples were then filtered through a 96-well plate with Sephadex (Sigma-Aldrich, Germany) by centrifugation (3180 × g, 3 min). In the next step, samples were separated electrophoretically in an ABI Prism 3130 Genetic Analyzer instrument (Applied Biosystems, USA). Electropherograms were analyzed with the Lasergene SeqMan Pro (version 12.2.0) software (DNASTAR, USA). Since both genes are located in close vicinity on ECA25, the linkage disequilibrium (LD) structure between the polymorphisms was described using HaploView software [25]. Thirty-nine SNPs from the PCH, PPH, and PWH breeds, posing sufficient genotype frequencies were selected for this analysis.

### Gene expression and composition of mare’s milk

This study was approved by the National Commission for Ethics of Animal Experimentation, and the local Ethics Committee for Animal Research (Poznan, Poland; permission number: 39/2012). During the entire experiment, the mares remained under veterinary control and did not manifest any symptoms of disease.

Mare milk samples used in this study came from four Polish national studs (Kobylniki, Sierakow, Racot, and Nowe Jankowice). The horses belonged to the three phylogenetically distinct breeds: PPH (n = 20), PWH (n = 27), and PCH (n = 27). All animals were kept under similar environmental conditions. Milk samples were collected manually in the morning (7–9 a.m.) three times during lactation (in the fifth, tenth and fifteenth weeks *postpartum*). During milking, the mares and foals remained in visual contact. After milk collection, 15 ml of the sample was frozen in liquid nitrogen for the gene expression studies, and the rest was stored at -20°C for the milk composition analysis. A total of 222 equine milk samples of 100 ml each were collected.

Total RNA from milk somatic cells (MSC) was obtained by extraction using TriPure Isolation Reagent (Roche, USA), according to a previously described procedure [26]. cDNA synthesis was performed with a Transcriptor High Fidelity cDNA Synthesis Kit (Roche, USA) according to manufacturer’s instructions. The standard curve based method was applied to *LGB1* and *LGB2* genes expression quantification, using real-time PCR technique. Firstly, the proper fragments of all analyzed genes were PCR amplified and visualized on 1.5% agarose gel stained with ethidium bromide. Each PCR product was extracted from the gel and purified using the GeneJET Gel Extraction Kit (Thermo Scientific, USA). After the DNA concentration assessment using Nanodrop 2000 system (Thermo Scientific, USA) a serial, 10-fold dilutions (standards) have been prepared. Afterwards, the standards were used as template for real-time PCR amplification, in order to create the appropriate standard curves. The relative transcript level measurements using a LightCycler 480 real-time PCR instrument (Roche, USA) were carried out in duplicate for the *LGB1* and *LGB2* genes and four reference genes (*ACTB*, *GAPDH*, *TOP2B*, and *KRT8*). Selection of internal control genes was conducted using the GeNorm and NormFinder tools, as previously described [26]. Primers and TaqMan probe sequences for real-time PCR were designed and synthesized by TIB MolBiol (Germany) (S1 Table). Each run of amplification contained the tested samples, proper DNA standard and the negative control (without cDNA). The following cycling conditions were applied: initial denaturation (95°C /5 min) followed by 45 cycles of amplification (95°C/10 s; 60°C/30 s; and 72°C/1 s). Afterwards samples were cooled (40°C/30 s). The results obtained for the *LGB1* and *LGB2* genes were normalized to the geometric mean of mRNA abundances of the above listed reference genes, in line with the protocol recommended by Vandesompele et al. (2002) [27].

The basic composition of mare’s milk as protein, fat, and lactose content was determined by automated infrared analysis in a Milkoscan FT2 instrument (Foss Electric, Denmark). The milk β–lactoglobulin content was established using an Agilent 1100 Series reverse phase high-performance liquid chromatograph (Agilent Technologies, Germany), following the methodology described by Puppel et al. (2016) [28]. LGB peaks were identified by comparison with BLGA Lot100M7026V and BLGB Lot048K7003V bovine standards (Sigma-Aldrich, USA). Separation was performed at room temperature using a solvent gradient on a Jupiter C18 300A column (Phenomenex, Torrance, USA) with chromatographic conditions as follows: solvent A was acetonitrile (Merck, Germany), water (Sigma-Aldrich, USA), and trifluoroacetic acid (Sigma-Aldrich, USA) in a ratio of 50:950:1 (v/v/v); solvent B was acetonitrile, water, and trifluoroacetic acid in a ratio of 950:50:1 (v/v/v); the total run time was 44 min, the flow rate was 1.2 mL min^-1^, and the detection wavelength was 220 nm; the injection volume of the final solution was 25 μl.

### Statistical analyses

After elimination of records with missing phenotypic data, the total number of 213 observations (derived from 73 animals) have been subjected to association studies (all tested genotypes and phenotypes are shown in S5 Table). Mixed ANOVA was used to determine the effect of the *LGB1* and *LGB2* gene polymorphisms on their relative transcript level in milk somatic cells, total milk β–lactoglobulin content, and the concentrations of the milk’s basic components (protein, fat, and lactose). We use a model incorporating the fixed effect of breed and sampling time (fifth, tenth, or fifteenth week *postpartum*) as a repeated-measure factor. The unknown variance components were estimated with the restricted maximum likelihood (REML) method [29]. Before final analysis, each factor included in the statistical model was tested using the Kruskall–Wallis and Friedman tests [30,31]. Obtained p-values were corrected for multiple testing using Tukey-Kramer adjustment. In the association study, only genotype groups spanning more than five phenotypic observations from the PPH, PWH, and PCH breeds were considered. Finally, 28 and 11 variants were selected for the *LGB1* and *LGB2* gene analysis, respectively. Additionally, the Spearmans’s correlation coefficient was calculated for *LGB1* and *LGB2* relative transcript abundances, milk LGB content and the total milk protein concentration. All statistical comparisons were conducted in SAS EG 7.1 software (SAS Institute Inc., USA).

## Results

### Equine *LGB1* and *LGB2* gene variants

The initial screening for polymorphisms with a multibreed panel of 96 horses revealed the presence of 83 variants (47 and 36 for *LGB1* and *LGB2* genes, respectively). Among them 81 were SNPs and 2 were InDels). Some of these were broadly distributed across the breeds (for example, the rs69324332 and rs394407776 *LGB1* SNPs were found in eleven of the twelve horse breeds), while some variants were detected in just one horse breed (e.g., the *LGB1* rs395074979 and rs397277457 SNPs, which were recorded exclusively in Silesian horses). In the case of the *LGB1* gene, only one SNP was previously unknown (a C>T substitution found in intron 5, chromosomal coordinates ECA25: 38875547). This SNP was detected in Percheron and Fjording horse breeds (S2 Table).

Analysis of the *LGB2* gene revealed the presence of 36 genetic variants. Most of them (29) were already present in databases, but seven (five SNPs and two InDels) were previously unknown. One novel polymorphism—a dinucleotide (GC) InDel (ECA25: 38896038–38896039)—was found in the 5’-flanking region of the *LGB2* gene. This variant has segregated in nine horse breeds (S2 Table). Five of the seven remaining novel variants (three SNPs and an InDel) were located in introns (3–5), and only two (an A>T substitution, ECA25: 38895282 and a C>G substitution, ECA25: 38891425) were found in the *LGB2* coding sequence (exons 1 and 5, respectively). The first of these (an A>T missense substitution, p.24Thr>Ser) was seen only in the Silesian breed, while the second (C>G missense substitution, p.174Gly>Arg) was recorded in the Fjording, Haflinger, Shetland Pony, Silesian, and Polish Warmblood Horse breeds. More details on the genetic variants are presented in S2 Table. The information about previously unknown polymorphisms was deposited in European Variation Archive (EVA) repository (project number: PRJEB35282).

**Fig 1:**
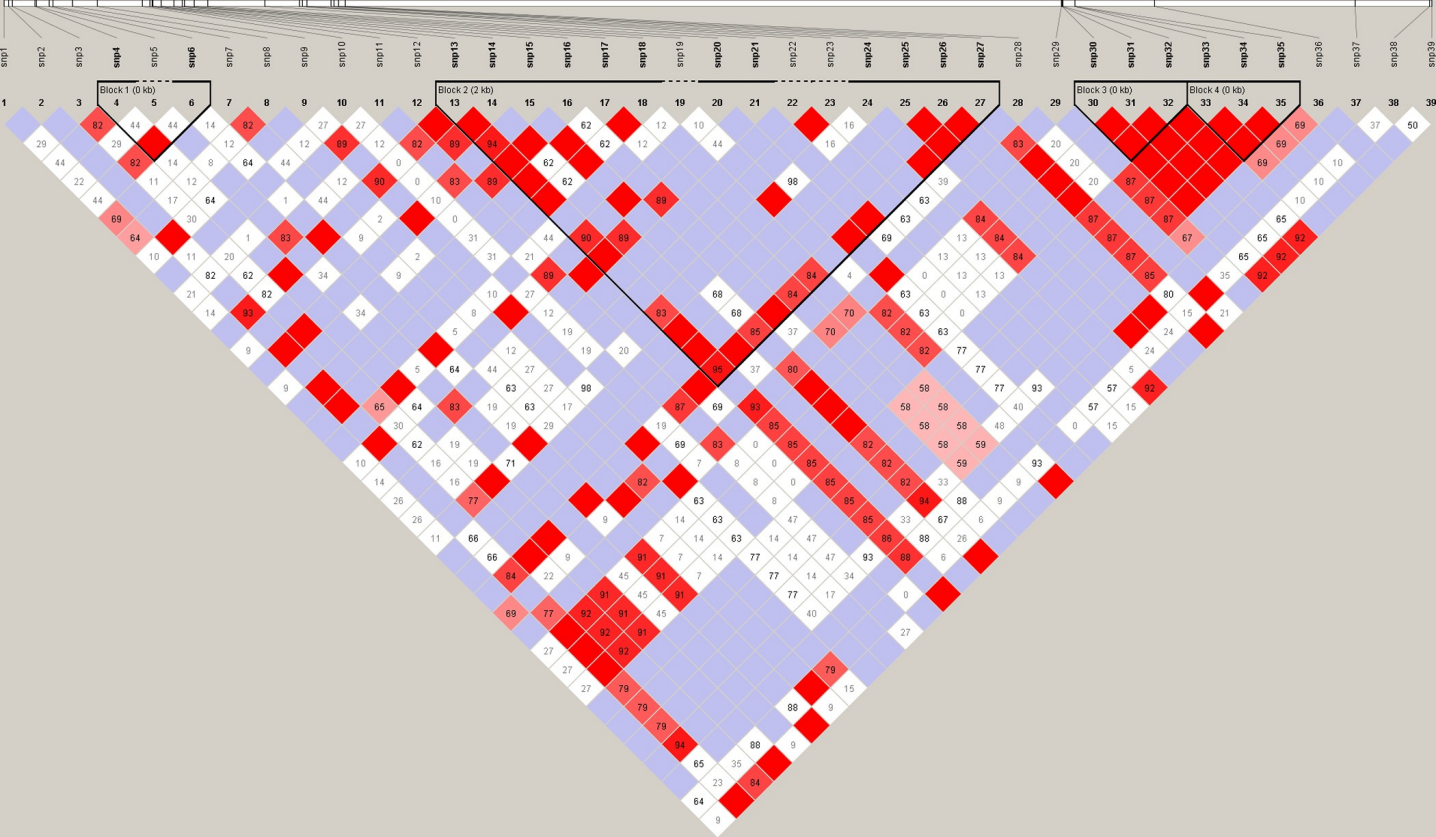
Linkage disequilibrium (LD) across the tested fragment of ECA25 (HaploView tool). Numbers in blocks denote D’ values. Red blocks without numbers show full LD between pairs of *loci*. Empty blue blocks denote a lack of LD between pairs of *loci*.

Analysis of linkage disequilibrium (LD) was performed for the 39 variants posing sufficient genotype frequencies and revealed the presence of four potential haplotype blocks (2 in *LGB1* and 2 in *LGB2* gene). The largest block spanned twelve polymorphisms of the *LGB1* gene (Fig 1). Generally, the LD between pairs of *loci* varied from almost none to full LD. However, it is worth noting that, in several cases, strong linkage was detected not only for SNPs located within the same gene, but also intragenic ones.

### Effect of horse breed and lactation period on the expression of the *LGB1* and *LGB2* genes

**Fig 2:**
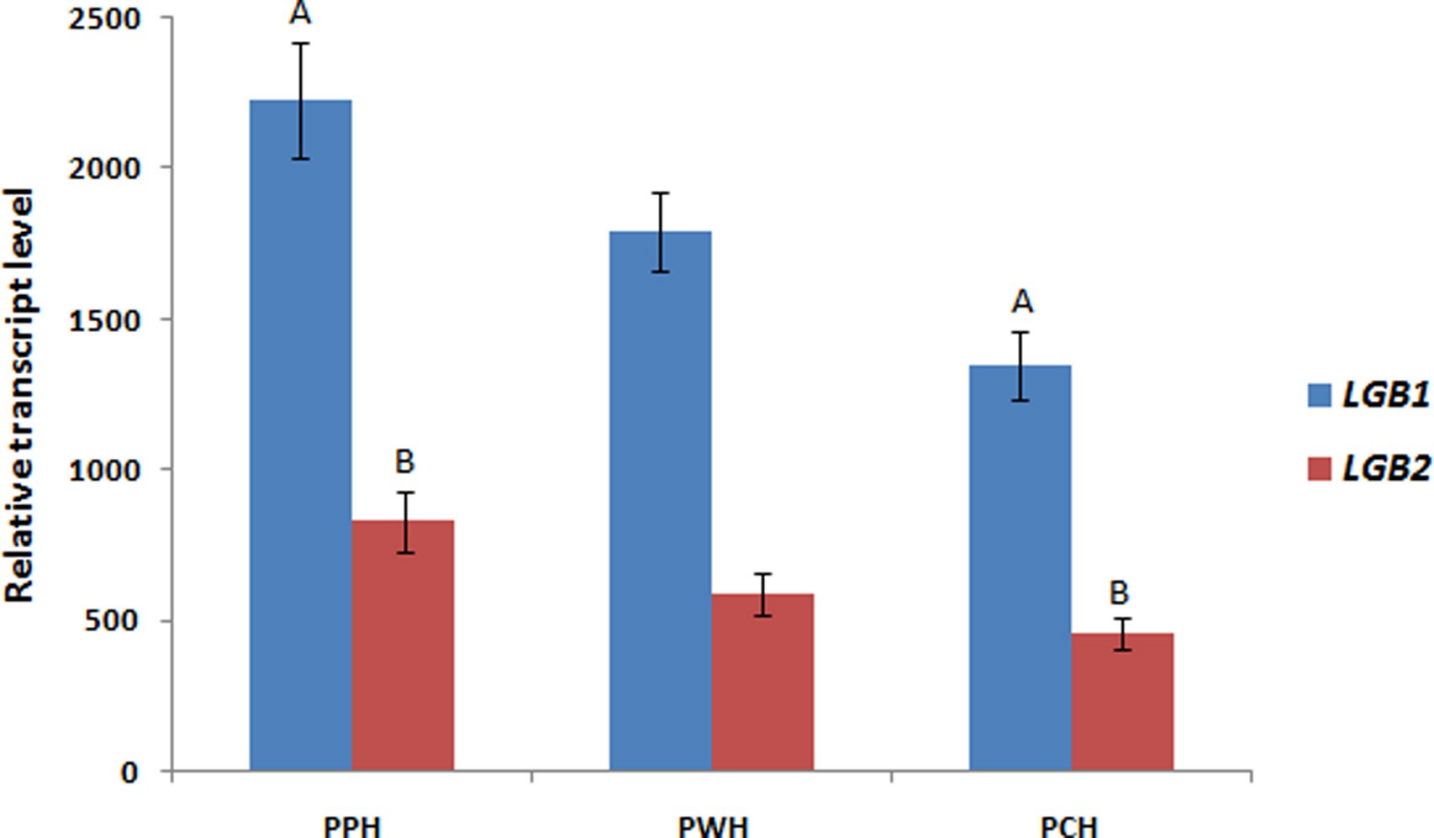
Interbreed comparison of *LGB1* and *LGB2* relative transcript levels. Values are means with standard errors. Means marked by the same uppercase letter (for each gene separately) differed significantly (p < 0.01).

Comparison of the relative transcript levels of *LGB1* and *LGB2* (measured in milk somatic cells) between the horse breeds showed the highest mRNA abundances of both genes in the PPH breed and the lowest in PCH (p < 0.01). The *LGB1* and *LGB2* transcript levels found in the PWH breed was intermediate, and did not differ significantly from these recorded for the two remaining breeds (p > 0.05) (Fig 2). Interbreed comparison of milk LGB protein content found the highest β-lactoglobulin concentration in the PWH breed and the lowest in milk derived from PPH mares (p < 0.01) (Table 1).

**Table 1:** Comparison of milk LGB concentration between the horse breeds and lactation time-points.

Lactation stage / horse breed	Number of milk samples	Average milk LGB conc. (g/L)	SEM
Week 5	70	2.93	0.08
Week 10	72	2.99	0.09
Week 15	70	2.83	0.09
PPH	59	2.73^A^	0.09
PWH	79	3.08^A^	0.09
PCH	75	2.89	0.08

SEM—standard error of the mean. Horse breeds acronyms: PPH—Polish Primitive Horse, PWH—Polish Warmblood Horse, PCH—Polish Coldblood Horse. Values marked by the same uppercase letter differed significantly (p < 0.01).

**Fig 3:**
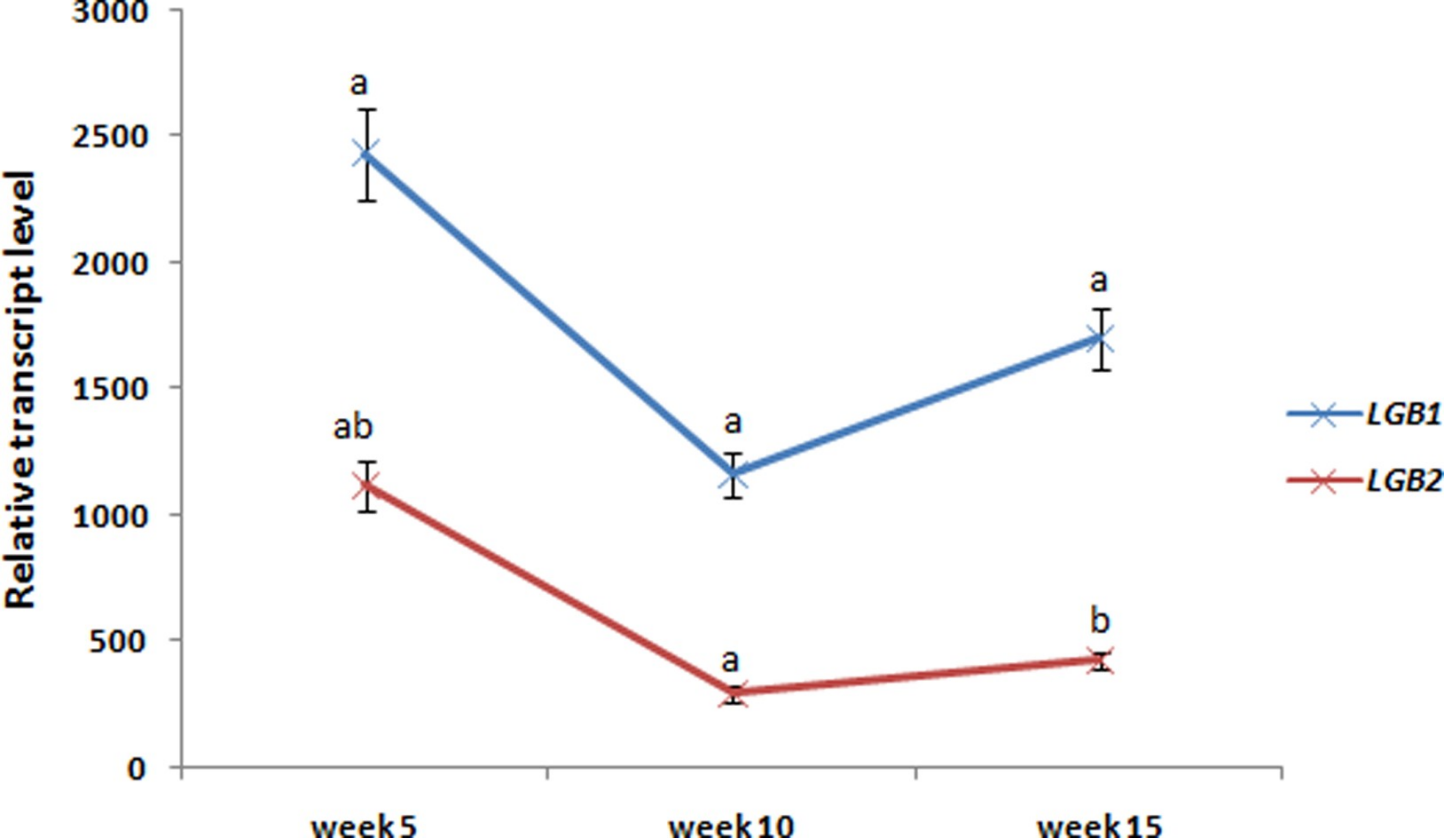
Changes in *LGB1* and *LGB2* genes relative transcript abundances across lactation time-points. Means marked by the same lowercase letter (for each gene separately) differed significantly (p < 0.05).

Tracing the expression profiles between the three lactation time-points (fifth, tenth, and fifteenth weeks *postpartum*) revealed similar trends for the *LGB1* and *LGB2* gene relative transcript levels. The highest mRNA abundances of both genes were recorded for the samples collected in the fifth week after foaling. The relative transcript level then decreased significantly (p < 0.05) between weeks 5 and 10. By the last time-point (week 15), mRNA levels had increased relative to week 10, but this elevation was only statistically significant for the *LGB1* transcript (p < 0.05) (Fig 3). A similar comparison for milk LGB concentrations showed that the highest β-lactoglobulin level occurred in week 10 of lactation and the lowest level at week 15; however, the recorded differences were not statistically significant (p > 0.05) (Table 1).

### Relationship between *LGB1* and *LGB2* gene variants, gene expression, and milk composition

The association analysis pointed to several statistically significant relationships between genotypes in the *LGB1* and *LGB2* variants, gene expression, and milk composition traits (S3 Table). The strongest relationship was seen for the two linked *LGB1* SNPs (rs1143515669 and rs1144647991) and the total milk protein content. The heterozygous horses (AG or AC) had the significantly (p < 0.01) higher protein concentrations than the homozygous genotype carriers (GG or AA, in rs1143515669 and rs1144647991 SNPs, respectively). This association was seen when the PPH breed was considered separately, as well as when all breeds were tested together. A similar, significant association was detected for the genotype in *LGB1* rs1140322039 SNP and the total milk fat concentration, when only the PWH breed was considered (p<0.01) and when all horse breeds were tested together (p < 0.05). The only relationship between genotype and milk β-lactoglobulin abundance was observed for the rs1140333438 polymorphism located in the *LGB1* gene. The GA genotype carriers had significantly (p < 0.05) higher milk LGB concentration than the GG homozygous mares, taking all breeds together. Moreover, the rs1150055140 variant was associated with *LGB1* (p < 0.05) and *LGB2* (p < 0.01) gene relative transcripts level, both when the PPH breed was considered separately and when all breeds were taken together; increased expression levels of both genes were seen for the CA genotype). We also recorded a significant relationship between *LGB2* rs1146870844 SNP and *LGB2* gene expression level (p < 0.01, PWH breed). Additionally, significant (p < 0.05) associations were observed between genotypes and several traits, namely milk lactose content (*LGB1* rs1151091548, PCH breed; *LGB2* rs1147750066, PPH breed), total milk protein concentration (three linked SNPs: *LGB1* rs1142388519, rs1143386853 and *LGB2* rs1138764575, PWH breed), *LGB1* gene relative transcript level (*LGB1* rs69324332 and rs69324336, PWH breed) and *LGB2* relative transcript level (two linked *LGB2* variants: rs1141488737 and 1144796536, PCH breed). All statistically significant comparisons are shown in detail in S3 Table.

## Discussion

Although our knowledge of the composition of mare’s milk and its beneficial impact on human health continues to expand, it is still not a widely used animal product, especially in European countries. Due to the small scale of production of mare milk in comparison to cow’s milk, equine milk remains relatively expensive and is used in the majority of cases as a health-promoting supplement rather than as a regular nutritional product. Equine milk is considered valuable, especially because of the significant amount of bioactive compounds it contains and its low allergenicity [3,4] It is thus often recommended for people who are hypersensitive to proteins found in ruminants’ milk and to those who suffer from other disorders (including skin problems, increased serum cholesterol level, hepatitis, and gastric ulcers) [5,32]. Despite the great similarity between the composition of mare’s milk and human breast milk, there are several notable differences. One of these is the presence of β-lactoglobulin, which is a very abundant protein in equine milk (and in the milk of many other mammalian species), yet is totally absent in human breast milk [4]. However, the LGB concentration of mare’s milk varies strongly between particular individuals, even considering only domestic horses. In the previous experiment of Markiewicz-Kęszycka et al. (2013) [13], the LGB concentrations fell within the range of 1.4–4.17 g/L, whereas in our present study, the variability was slightly higher (1.2–4.9 g/L). This may be associated with the fact that Markiewicz-Kęszycka et al. (2013) [13] considered only the Polish Coldblood Horse breed, and all samples were collected at a late lactation stage; in the present study, samples were derived from three horse breeds (PPH, PCH, and PWH) at weeks 5, 10, and 15 after foaling.

The initial screening for polymorphism in the panel of twelve horse breeds has revealed the presence of 83 polymorphic sites. Majority of them (47) were located in the *LGB1* gene, however all the missense SNPs (6) were detected exclusively in the *LGB2* gene coding sequence. This is generally in agreement with the previously published work by Brinkmann et al. (2016) [11] who described the presence of eight nonsynonymous SNPs in the *LGB2* gene and only a one missense variant within the *LGB1* gene (sequence encoding the LGB1 signal peptide).

Taking into consideration the high estimates of heritability (h^2^ = 0.8) found for Holstein–Friesian cattle milk LGB content [33], it would seem clear the observed intrabreed and interbreed variation in the β-lactoglobulin content of equine milk is also related to genetic factors (such as polymorphism and the expression of *LGB* genes). However our results suggest that the regulation of equine *LGB* gene expression is highly complex. Although we have described several statistically significant relationships between genotypes and the mRNA levels of both gene measured in milk somatic cells, only one polymorphism (rs1140333438) of *LGB1* revealed an association with the milk β-lactoglobulin content. Unexpectedly, this particular SNP did not affect the relative transcript level of the *LGB1* gene (p > 0.05). Generally, when we measure the variability of *LGB1* and *LGB2* gene expression profiles at the mRNA stage, we can note that they do not correspond to the variation in the amount of milk β-lactoglobulin (for example, the highest relative transcript abundance of both genes was noted for the PPH breed, while the average β-lactoglobulin content in PPH mares milk was low) (Fig 2 and Table 1). Similar results were obtained in our studies of other genes encoding equine milk proteins [20,21,34]; this confirms the complexity of the mechanisms responsible for regulating gene expression. This complexity frequently leads to a lack of linear relationship between mRNA abundance and the final amount of protein [35]. Interestingly, we observed similar trends in the mRNA levels of both genes across the three lactation time-points (Fig 3), which may suggest the existence of coregulatory mechanisms of expression. This hypothesis is partly supported by the fact that, in the case of one SNP (rs1150055140), a statistically significant relationship was noted with the expression levels of both genes. Moreover, we have recorded the highly significant correlation (r^2^ = 0.83, p < 0.01) of both genes relative transcript abundances (S4 Table). However further study is needed to fully uncover the mechanisms that influence equine *LGB1* and *LGB2* gene expression patterns. Taking these results into account, we must underline that, due to the lack of simple relationship between transcript and protein levels, the experiments investigating gene expression should perhaps be conducted for both the mRNA and protein stages. When this is not done, conclusions should be drawn very carefully. For example, looking at the relative transcript abundance of both *LGB* genes, we can assume that the main contribution to mare’s milk β-lactoglobulin concentration is played by the *LGB1* gene (as we noted greater expression of this gene than of *LGB2* in all lactation stages). However, when we consider the lack of simple association between mRNA and final protein expression observed in our study, this assumption no longer appears reliable. In the present study, tracing the connections between transcripts and protein level was also challenging due to the lack of HPLC standards for equine β-lactoglobulin isoforms (the corresponding bovine standards were used). We were therefore unable to distinguish between *LGB1* and *LGB2* proteins, and only the total beta β-lactoglobulin concentration was taken into account. Since the technical variation is considered as one of the most important factors influencing the reliability of transcript and protein levels correlation estimation [36], we cannot exclude that in the case of the present study the “technical noise” was the main reason of the recorded lack of simple association between LGB genes mRNA abundances and milk β-lactoglobulin content. Therefore further investigations based upon the larger number of samples and other molecular methods are needed to fully confirm or undermine the obtained results. This is justified especially if we take into account that previous studies considering the global gene expression in mammalian cells have shown that although the final amount of protein depends mainly on translation stage control, still about 40% of variability can be explained by the variation of mRNA level [37].

From the methodological point of view, the lack of simple relationship between the tested genes transcript levels and the milk LGB concentration may also be related to the applying of non-fully homogenous source of RNA (milk somatic cells) for gene expression analyses. It is well known that the population of MSC is consisted of various cell types, including the mammary epithelial cells (crucial for the milk proteins expression) and leukocytes (lymphocytes, macrophages and polymorphonuclear neutrophils) [38]. Their proportion may vary between milk of particular individuals which depends (among others) on a current immunological status of the mammary gland. Since in the present study we were not able to separate the mammary epithelial cells from the other MSC types, we can suppose that the measurements of gene expression are not fully accurate. On the other hand, the previous investigations on ruminants have clearly shown that the MSC populations is a very good source of RNA for gene expression level assessment, reflecting precisely the transcriptional status of the mammary gland [39]. It should also be emphasized that to avoid a potential bias related to the variability of mammary epithelial cells number in particular samples, we used the *KRT8* (Keratin-8) as one of the reference genes for real-time PCR results normalization. Although this is not the classical housekeeping gene, due to its specific expression in milk-secreting cells, *KRT8* can be used for normalization of mammary epithelial cells number variability observed between particular milk samples [26,40].

Based on published studies of ruminants [16,17,41], we have assumed that equine *LGB* gene variants can serve as valuable markers of equine milk composition, including LGB content and gross milk composition traits. The most repeatable association recorded in our study was the relationship between particular SNPs (such as *LGB1* rs1143515669, rs1144647991 and rs1140322039) and basic milk composition traits (total milk protein or total milk fat concentration). It seems that selected equine *LGB* gene variants can be considered potential markers for these particular traits; however, further studies with larger animal groups of different horse breeds are necessary. Associations between β-lactoglobulin polymorphisms and total milk protein and/or fat concentration have previously been described for cattle [12,42,43] and sheep [44,45]. We have also recorded a relationship between genotypes in rs1151091548 *(LGB1)* and rs1147750066 *(LGB2)* SNPs and the milk lactose level of PPH and PCH mares, respectively. This association may prove interesting from the consumer point of view (in terms of effects on milk taste, lactose intolerance, etc.) as well as in terms of the processing of mare’s milk (such as fermented products making); studies of equine *LGB1* and *LGB2* gene variants should therefore be continued.

Published reports on the genes that encode milk proteins have concentrated predominantly on variants detected within coding regions and on their potential effect on amino acid sequences. However, our current knowledge suggests that polymorphisms affecting gene expression or milk composition may be located in various parts of the gene or even intergenically. For example, the genetic variation within introns can introduce novel splice sites or affect transcript stability [46]. There are also many experiments showing relationships between polymorphisms in regulatory regions, gene expression, and milk composition. In the case of the gene encoding bovine β-lactoglobulin, it has been shown that some 5’-flanking variants (g.-731G>A and g.-215C>A) are associated with expression variability measured on the protein or mRNA level [47,48].

For these reason, we decided to analyze the entire nucleotide sequence of equine *LGB1* and *LGB2* genes. Although we have described some promising associations between genetic variants, gene expression and milk composition traits, we were unable to investigate all the detected polymorphisms (including several novel ones) due to their insufficient frequencies in the PPH, PCH, and PWH breeds. Our study should thus be treated as a good starting point for broader analyses of *LGB1* and *LGB2* gene polymorphism, expression patterns, and impact on the variability of mare’s milk composition.

## Supporting information

S1 Table PCR primer sequences and other amplification details. (DOCX)

S2 Table Detected *LGB1* and *LGB2* variants. MAF–minor allele frequency. Horse breeds acronyms: FJOR–Fjording, HUC–Hucul, HAFL–Haflinger, PCH–Polish Coldblood Horse, PPH–Polish Primitive Horse, PWH–Polish Warmblood Horse, SHET–Shetland Pony, SIL–Silesian, THOR–Thoroughbred, WELP–Welsh Pony, ARAB–Arabian, PERCH–Percheron.
(XLSX)

S3 Table Association studies results. SNP—single nucleotide polymorphism, RA—relative abundance, SEM—standard error of the mean, n—number of horses, k—number of phenotypic observations. Within column (for each SNP separately) means marked with the same superscript differed significantly at ^a^p<0.05 or ^A^p<0.01.
(XLSX)

S4 Table Spearman’s correlation coefficients. Statistically significant correlations are marked with the superscripts: ^a^p<0 .05 or ^A^p<0 .01.
(DOCX)

S5 Table Genotypes and phenotypes (gene expression and milk composition traits measurements) used in association study. Horse breeds acronyms: PPH—Polish Primitive Horse, PWH—Polish Warmblood Horse, PCH—Polish Coldblood Horse.
(XLS)
