# Supplementary material for: Genes encoding equine β-lactoglobulin (LGB1 and LGB2): Polymorphism, expression, and impact on milk composition
Source: PLoS One. 2020 Apr 22;15(4):e0232066. doi: 10.1371/journal.pone.0232066 (PMC7176115; doi:10.1371/journal.pone.0232066)
Supplement: S1 Table — (DOCX) [file pone.0232066.s001.docx]

***S1 Table. PCR primer sequences and other amplification details.***

| **Screening for polymorphism and genotyping** | | | | | |
| --- | --- | --- | --- | --- | --- |
| **(Sanger sequencing)** | | | | | |
| **Primer name** | | **Primer sequence** | **PCR product size (bp)** | **Primer annealing temp. (°C)** | **Polymerase** |
| *LGB1_5'-flanking_IF* | | TGT GCC CCA TGT GGG ACA GAC G | 670 | 65 | DreamTaq® (ThermoScientific) |
| *LGB1_5'-flanking_IR* | | CCC CGA CCT CAC AGC CTC CTC TAT |  |  |  |
| *LGB1_5'-flanking_IIF* | | AGC GAG ATG TGC TCT TCT GCG ACT GG | 649 | 65 | DreamTaq® (ThermoScientific) |
| *LGB1_5'-flanking_IIR* | | ATG GGG CAC AGA TCA TTG GCA CCA C |  |  |  |
| *LGB1_fr_1_F* | | CTC AGA GTG CAC CCA CAG C | 612 | 65 | FIREPol® (Solis BioDyne) |
| *LGB1_fr_1_R* | | AAA AAT GTT GGG TGG CAG AG |  |  |  |
| *LGB1_fr_2_F* | | GGG CAT TAT TTA GCC CAT ATC A | 606 | 65 | FIREPol® (Solis BioDyne) |
| *LGB1_fr_2_R* | | CAC TCA CAG CAT AGC CAG GTC |  |  |  |
| *LGB1_fr_3_F* | | GGA GAT CAT CCT GCG TGA AG | 628 | 65 | FIREPol® (Solis BioDyne) |
| *LGB1_fr_3_R* | | TGG ACA CCA TTC TGA TAA AGT CA |  |  |  |
| *LGB1_fr_4_F* | | CAG CTG TTA GAG GTG ACA GTG G | 653 | 65 | FIREPol® (Solis BioDyne) |
| *LGB1_fr_4_R* | | AAC GAG AGG ATG AGT GAA TGA ATC |  |  |  |
| *LGB1_fr_5_F* | | CTC AGG AAG CAT CTG TGT GGT | 601 | 65 | FIREPol® (Solis BioDyne) |
| *LGB1_fr_5_R* | | CAG AGT GGT TGA GAA ATG AGG TC |  |  |  |
| *LGB1_fr_6_F* | | GAT CAG TGT GGA GAC CTC ATT TC | 674 | 63 | FIREPol® (Solis BioDyne) |
| *LGB1_fr_6_R* | | GAA CAA AGC CTG TTG GAT TCA TA |  |  |  |
| *LGB1_fr_7_F* | | CTG GTG ATG CAG AGC AGA CAC | 620 | 65 | FIREPol® (Solis BioDyne) |
| *LGB1_fr_7_R* | | GAA TTT CTC CAT GAT CTC CTC ATC |  |  |  |
| *LGB1_fr_8_F* | | AGA TGG TCG ATG AGG AGA TCA T | 621 | 65 | FIREPol® (Solis BioDyne) |
| *LGB1_fr_8_R* | | CAG AAG TAG TGG TGG GGA CAT AA |  |  |  |
| *LGB1_fr_91_F* | | GGA TGT TGT CAC CTG TGT CCT | 610 | 65 | FIREPol® (Solis BioDyne) |
| *LGB1_fr_9_R* | | GTC TCC TTC CGG AAA GTT TAA GAG |  |  |  |
| *LGB2_5'-flanking_IF* | | GCC AGG CCG AGT CTA ACG GGA TGG | 703 | 65 | DreamTaq® (ThermoScientific) |
| *LGB2_5'-flanking_IR* | | GCG GCC AGC CGG GAC ACG |  |  |  |
| *LGB2_5'-flanking_IIF* | | GTC AAC GCC CTC GAC GGT TCT CTA | 662 | 65 | DreamTaq® (ThermoScientific) |
| *LGB2_5'-flanking_IIR* | | CCC CTG GGG CCT GGA CAG TTC |  |  |  |
| *LGB2_fr_1_F* | | CTC TCC AGC CTC CCT CCT TTA T | 667 | 65 | FIREPol® (Solis BioDyne) |
| *LGB2_fr_1_R* | | CAG CTC CTC GAC GTA CAC TCT |  |  |  |
| *LGB2_fr_2_F* | | GAG AGT CCA CTG TGG GTC TGG | 614 | 65 | FIREPol® (Solis BioDyne) |
| *LGB2_fr_2_R* | | CAG CTC AAA ACA GAC CCT GTC TA |  |  |  |
| *LGB2_fr_3_F* | | GAC GTC CTT GGA AAT GCT GT | 697 | 65 | FIREPol® (Solis BioDyne) |
| *LGB2_fr_3_R* | | CCC GCT CTC TTC CTA TGT CA |  |  |  |
| *LGB2_fr_4_F* | | AGT TGT GCA GGC ACT CTC G | 603 | 65 | FIREPol® (Solis BioDyne) |
| *LGB2_fr_4_R* | | CAG GGA CAG GGG CTA AGG |  |  |  |
| *LGB2_fr_5_F* | | GTG AGT GTG GTG GGT ACA ATC TT | 646 | 65 | FIREPol® (Solis BioDyne) |
| *LGB2_fr_5_R* | | AAG TGT TAT GGA CCC CAG AGG |  |  |  |
| *LGB2_fr_6_F* | | GGA GTG CCA ACA CAT CGA G | 605 | 61.4 | FIREPol® (Solis BioDyne) |
| *LGB2_fr_6_R* | | AAG TCA CCC ACA TCA GAG CAC |  |  |  |
| *LGB2_fr_7_F* | | AAC AAC GAG CAC TCA TGA CGA C | 643 | 65 | FIREPol® (Solis BioDyne) |
| *LGB2_fr_7_R* | | CTG AGG TCG GTA GAG CTT GGT C |  |  |  |
| *LGB2_fr_8_F* | | GGC CAT TTT CCT ACC ATA ACT G | 683 | 65 | FIREPol® (Solis BioDyne) |
| *LGB2_fr_8_R* | | GTT GGT ACG TTA CCC GAT GAG |  |  |  |
| *LGB2_fr_9_F* | | ATT GCT GGT TCT GTG GGA AC | 637 | 65 | DreamTaq® (ThermoScientific) |
| *LGB2_fr_9_R* | | CTG TGA TTC CTG GGG AAC CT |  |  |  |
| *LGB2_fr_10_F* | | CAG AGC GAT GCG GTT TCT A | 634 | 65 | DreamTaq® (ThermoScientific) |
| *LGB2_fr_10_R* | | GCA CTC AGG TTT ATT TCT TTA CTG C |  |  |  |
| *LGB2_fr_11_F* | | CTC ATC GGG TAA CGT ACC AAC | 614 | 65 | FIREPol® (Solis BioDyne) |
| *LGB2_fr_11_R* | | GAA AGA CTC AGG CAG AGA GAG C |  |  |  |
| **Relative transcript level analysis** | | | | | |
| **(Real-time PCR)** | | | | | |
| **Primer name** | **Primer sequence** | | **PCR product size (bp)** | **Primer annealing temp. (°C)** | **Polymerase** |
| *LGB1RT_F* | CTG CGT GAA GGG GAG AAC A | | 100 | 60 | LightCycler® 480 Probes Master (Roche) |
| *LGB1RT_R* | TGT CCT CAT CCA GGT AGT TGA TCT TG | |  |  |  |
| *LGB1probe* | AGA AAG ACT GAG AGC CCA GCG GA | |  |  |  |
| *LGB2RT_F* | TGT GTT CAC GGT CAA CTA TCA AGG | | 119 | 60 | LightCycler® 480 Probes Master (Roche) |
| *LGB2RT_R* | ACA CCA TGC CGT GCT CAG | |  |  |  |
| *LGB2probe* | TGG ACA CAG ACT ACG CCC ACT ACC ATGT | |  |  |  |
| *ACTBRT_F* | TCC TTC CTG GGG CAT GGA ATC | | 146 | 60 | LightCycler® 480 Probes Master (Roche) |
| *ACTBRT_R* | TCC TGT CGG CGA TGC CT | |  |  |  |
| *ACTBRTprobe* | CCG TAA GGA CCT GTA CGC CAA CAC AGT | |  |  |  |
| *KRT8RT_F:* | ACC CAG GAG AAG GAG CAG AT | | 108 | 60 | LightCycler® 480 Probes Master (Roche) |
| *KRT8RT_R:* | GCT CCA CTT GGT CTC CAG AA | |  |  |  |
| *KRT8RTprobe:* | GCA TCT GGA ACA GCA GAA CA | |  |  |  |
| *TOP2BRT_F:* | GCC AGC TGA CAA TAA ACA GAG G | | 101 | 60 | LightCycler® 480 Probes Master (Roche) |
| *TOP2BRT_R:* | TGC CTT TCC CAT TAT TCC AA | |  |  |  |
| *TOP2Bprobe:* | TTG ATC CTG AAT CTA ACA TTA TAA GCA | |  |  |  |
| *GAPDHRTF:* | GAG GAC CAG GTT GTC TCC TGC | | 101 | 60 | LightCycler® 480 Probes Master (Roche) |
| *GAPDHRTR:* | ATG AGC TTG ACA AAG TGG TCG TT | |  |  |  |
| *GAPDHRTprobe:* | ACC CAC TCT TCC ACC TTC GAT GCT | |  |  |  |
