# Supplementary material for: Genes encoding equine β-lactoglobulin (LGB1 and LGB2): Polymorphism, expression, and impact on milk composition
Source: PLoS One. 2020 Apr 22;15(4):e0232066. doi: 10.1371/journal.pone.0232066 (PMC7176115; doi:10.1371/journal.pone.0232066)
Supplement: S4 Table — Statistically significant correlations are marked with the superscripts: ap<0 .05 or Ap<0 .01. (DOCX) [file pone.0232066.s004.docx]

***S4 Table. Spearman’s correlation coefficients.***

| **r^2^** | **LGB1**  **mRNA** | **LGB2**  **mRNA** | **Milk LGB**  **conc.** | **Total milk**  **protein conc.** |
| --- | --- | --- | --- | --- |
| **LGB1**  **mRNA** | 1 |  |  |  |
| **LGB2**  **mRNA** | 0.83^A^ | 1 |  |  |
| **Milk LGB**  **conc.** | -0.03 | -0.02 | 1 |  |
| **Total milk**  **protein conc.** | 0.16^a^ | 0.23^A^ | 0.20^A^ | 1 |

Statistically significant correlations are marked with the superscripts: ^a^ p<0.05 or ^A^ p<0.01^.^
